# Wheat Proteomics for Abiotic Stress Tolerance and Root System Architecture: Current Status and Future Prospects

**DOI:** 10.3390/proteomes10020017

**Published:** 2022-05-22

**Authors:** Tanushree Halder, Mukesh Choudhary, Hui Liu, Yinglong Chen, Guijun Yan, Kadambot H. M. Siddique

**Affiliations:** 1 UWA School of Agriculture and Environment, The University of Western Australia, 35 Stirling Highway, Crawley, WA 6009, Australia; hui.liu@uwa.edu.au (H.L.); yinglong.chen@uwa.edu.au (Y.C.); guijun.yan@uwa.edu.au (G.Y.); 2 The UWA Institute of Agriculture, The University of Western Australia, 35 Stirling Highway, Crawley, WA 6009, Australia; 3 Department of Genetics and Plant Breeding, Faculty of Agriculture, Sher-e-Bangla Agricultural University, Dhaka 1207, Bangladesh; 4 ICAR-Indian Institute of Maize Research, Ludhiana 141001, India

**Keywords:** yield, stress-response, genes, root, proteins, enzymes

## Abstract

Wheat is an important staple cereal for global food security. However, climate change is hampering wheat production due to abiotic stresses, such as heat, salinity, and drought. Besides shoot architectural traits, improving root system architecture (RSA) traits have the potential to improve yields under normal and stressed environments. RSA growth and development and other stress responses involve the expression of proteins encoded by the trait controlling gene/genes. Hence, mining the key proteins associated with abiotic stress responses and RSA is important for improving sustainable yields in wheat. Proteomic studies in wheat started in the early 21st century using the two-dimensional (2-DE) gel technique and have extensively improved over time with advancements in mass spectrometry. The availability of the wheat reference genome has allowed the exploration of proteomics to identify differentially expressed or abundant proteins (DEPs or DAPs) for abiotic stress tolerance and RSA improvement. Proteomics contributed significantly to identifying key proteins imparting abiotic stress tolerance, primarily related to photosynthesis, protein synthesis, carbon metabolism, redox homeostasis, defense response, energy metabolism and signal transduction. However, the use of proteomics to improve RSA traits in wheat is in its infancy. Proteins related to cell wall biogenesis, carbohydrate metabolism, brassinosteroid biosynthesis, and transportation are involved in the growth and development of several RSA traits. This review covers advances in quantification techniques of proteomics, progress in identifying DEPs and/or DAPs for heat, salinity, and drought stresses, and RSA traits, and the limitations and future directions for harnessing proteomics in wheat improvement.

## 1. Introduction

Wheat (*Triticum aestivum* L.) is an important economic crop that ranks second in production, next to corn [1]. Wheat consumption per capita as food and feed is estimated to increase, especially in developing countries, transition economies, and industrial countries, as evident from the forecasted demand for import of 160 million metric tons in developing countries by 2030 [2]. Hence, considering the increase in global population by 25% (10 billion) by 2050 [3], world wheat production needs to double from current production (775.6 million tons) [1,4,5]. This target needs to be achieved under the increased frequency and intensity of various abiotic stresses, including heat, salinity, and drought under changing climate [6]. For example, drought stress reduces global wheat yields by 32% [7]. Recently, an agricultural production system simulator wheat model predicted yield loss will increase due to heat stress of −0.6 to 4.2% from 2071–2100 on the North China Plain (contributing 50% of wheat production in China) [8]. In addition, salinity is contaminating soil [9], reducing wheat yields by 40% [10].

Root system architecture (RSA) is a complex three-dimensional structure of in situ distribution of the root system within the rooting volume. It exhibits a specific spatial and temporal configuration of different root types [11]. RSA is the primary plant part to supply nutrients and water from the soil, and good anchorage in any environmental condition [12,13]. Therefore, RSA plays a key role in ensuring sustainable yields under different environmental scenarios. However, RSA is most affected by drought, salinity and heat stresses [14,15,16]. Therefore, improved RSA is important for stress tolerance improvement. However, root analysis in the soil as non-invasive imaging of root growth is difficult because it is laborious, time-consuming, hard to monitor and costly. Root sampling disturbs the soil habitat and root parts, e.g., root hairs, are often lost during RSA measurements. Moreover, field experiments are challenging due to uncontrolled environmental conditions [17,18,19,20]. However, the recent development of the semi-hydroponic systems enables high-throughput phenotyping of root morphological traits with non-destructive measurements of root growth during the vegetative stage [21].

**Figure 1:**
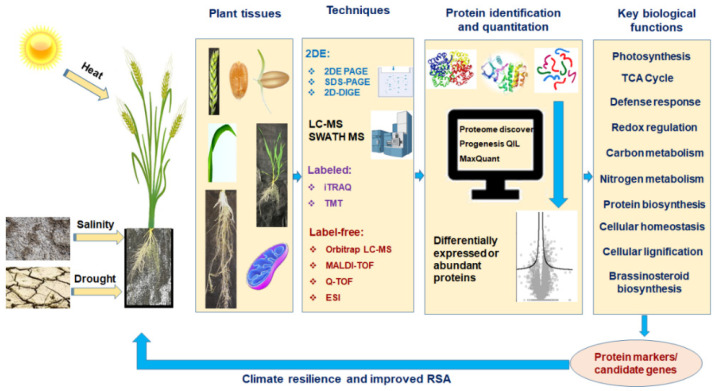
General workflow of proteomic approach to identify protein markers or protein-encoding candidate genes in wheat for heat, salinity, and drought stress, and root system architecture (RSA) improvements. Different plant tissues are used to extract proteins using various techniques, and to quantify them using relevant software to identify differentially expressed proteins or abundant proteins. Identified proteins together with their biological functions of stress tolerance and RSA, can be used as protein markers or genetic markers (genes that encode those proteins) development for marker-assisted breeding or genetic engineering; 2DE = two- dimensional gel electrophoresis; 2DE-PAGE = 2D polyacrylamide gel electrophoresis; 2D-DIGE = 2D difference gel electrophoresis; SWATH-MS = sequential window acquisition of all theoretical fragment ion spectra mass spectrometry; iTRAQ = isobaric tags for relative and absolute quantitation; TMT = tandem mass tag; MALDI-TOF = matrix-assisted laser desorption/ionization- time-of-fight; Q-TOF = quadrupole- time-of-fight and ESI = electrospray ionization. Images of LC-MS, a protein structure, wheat plant and mitochondria are modified from different sources [41,42,43,44].

Plant responses to abiotic stress and RSA are complex quantitative traits [22,23]. The molecular mechanisms and pathways for stress tolerance in crops can be determined using genomics, transcriptomics and proteomics approaches. Proteomics (study of the proteome, i.e., complete protein content of a genome) is the preferred approach as the end product for plant phenotypic expression (proteins) is the outcome of complex metabolic processes. Furthermore, plant growth and development involve a complex regulatory network and tissue-specific proteins [24,25,26,27]. Additionally, stress tolerance results from post-translational modification (PTM), a feature missing in other omics approaches [28]. It is important to understand the biochemical pathways of the trait behavior under different environmental conditions through robust protein profiling, PTMs of protein, and protein–protein interactions [29]. Proteomics involves (1) protein separation and identification through two-dimensional gel (2-D gel) or liquid chromatography-mass spectrometry (LC-MS) or coupled gel-free LC-MS (shotgun method: label-free and label-based); (2) detection of protein functions in the metabolic and signaling pathways using protein–protein interactions and characterizing PTMs, and (3) use of databases of model and non-model plant species and bioinformatic tools for candidate peptides [30,31] (Figure 1). However, as wheat has a large genome size (17 Gb) and highly repetitive genomes due to its hexaploid nature [32], the complex protein database of wheat is lacking [33]. Furthermore, the presence of complex compounds in root tissues hinders complete protein identification [24,34] and detecting low abundance proteins with an important role in stress tolerance and RSA remains challenging [35]. However, due to advances in MS, several proteins have been identified for heat, salinity, and drought stress tolerance, and RSA, which can serve as protein markers to improve RSA and/or abiotic stress tolerance in wheat. There are several reviews on advances in wheat proteomics for combating various abiotic stresses and root improvement [24,36,37,38,39,40]. Therefore, this review mainly focuses on the wheat proteomic journey; the potential and limitations of wheat proteomics and recent (last decade) advances in wheat proteomics for abiotic stresses, such as heat, salinity and drought, and RSA.

## 2. Proteomics: Edge over the Other Omics Techniques

Understanding the abiotic stress responses of crops is essential for addressing yield losses through breeding climate resilient cultivars. ‘Omics’ approaches, including genomics, transcriptomics, and proteomics, have contributed immensely to advancing research on the abiotic stress tolerance of plants [45]. The enormous data generated from these techniques allow for the investigation of complex regulatory mechanisms in plant metabolism under abiotic stress conditions [46]. Forward and reverse genetic approaches and genome-wide analyses conducted in model plants exhibiting multistress tolerance, such as *Arabidopsis thaliana* and the extremophyte relative *Thellungiella salsuginea* helped to reveal the underlying molecular mechanisms for stress tolerance [47]. Even though genomics and transcriptomics have generated vast knowledge on plant stress responses, the poor correlations between mRNA expression levels and protein abundance levels are a concern [48,49]. For example, Pan et al. [50] reported poor correlation patterns between the transcription and translation of the differentially abundant proteins (DAPs), indicating the importance of studying stress-responsive changes at the proteomic level. Furthermore, an integrative transcriptomic and proteomic study reported that 30% of transcribed proteins did not correspond to the translated root proteins in wheat under drought stress [51]. Systematic stress responsive patterns in root and shoot tissues were observed at proteomic levels, whereas the leaf and root transcriptomes showed distinct responsive patterns under salinity and drought stress [28]. Such deviations may be because the genome is static and thus does not provide a snapshot of an organism’s metabolism at a particular developmental time point [52]. As a technique, proteomics has advantages over other omics techniques, such as genomics and transcriptomics as it deals with key players maintaining cellular homeostasis [49,53]. 

## 3. Protein to Proteomics: Revisiting the Journey

**Figure 2:**
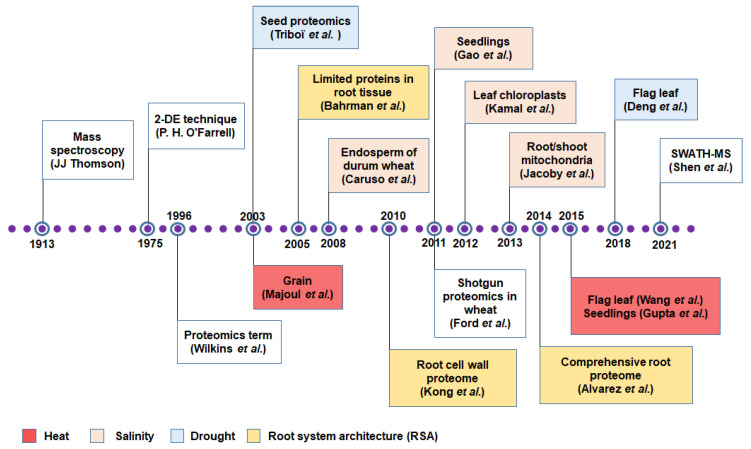
Journey of proteomics progress in terms of techniques and first studies in different plant parts of wheat under heat, drought and salinity stresses, and for root system architecture (RSA). The journey has been identified from a number of publications [56,57,58,59,60,61,62,63,64,65,66,67,68,69,70,71,72].

The term ‘protein’ originated from the Greek word ‘proteios’ (‘the first rank’) which was given to the molecules by Berzelius in 1838 [54,55]. The term ‘proteomics’ was first used in 1996 to denote the ‘PROTein complement of a genOME’ (Figure 2) [56]. ‘Proteome’ is defined as the overall protein content of a cell characterized by the localization, PTMs, interactions, and, turnover at a given time [54]. The earliest conventional proteomic technologies used to characterize biological samples included ion exchange chromatography, size exclusion chromatography (SE), affinity chromatography, enzyme-linked immunosorbent assays (ELISA), western blotting SE, affinity chromatography, and western blotting [54]. The field of proteomics has witnessed significant improvements in terms of accuracy, sensitivity, speed and the development of powerful analytical software.

This section mainly covers the evolution of different quantitative proteomic techniques, their potential, limitations and applications in plants. The journey of proteomics includes the discovery or use of important techniques, the first studies (to our best knowledge) of wheat proteomics under heat, salinity, and drought stresses and for RSA using different wheat tissues are represented in Figure 2. Generally, quantitative proteomic techniques are classified into ‘gel-based’ and ‘gel-free’ methods; the latter can be divided into ‘label-based’ and ‘label free’ depending on the labeling approach (Figure 1). After successful 2-DE in the early 1970s [57] (Figure 2), most proteomics research used this technique for protein identification [73]. However, this early gel-based conventional proteomic technique was limited to analyzing relatively few proteins at once and unable to analyze protein expression levels [74,75]. Advanced 2-DE techniques can now visualize thousands of proteins including multiple spots of different forms of the same proteins in a single gel [76], but it is labor intensive and time-consuming [77].

After first being discovered by Sir J. J. Thomson [58,78] (Figure 2), MS has been used widely in proteomics. Despite having low reproducibility, 2D polyacrylamide gel electrophoresis (2DE-PAGE) combined with MS remains a predominant methodology in plant proteomics. The technique has been modified into 2D difference gel electrophoresis (2D-DIGE) to offer higher reproducibility and greater sensitivity [79,80]. Nevertheless, the 2-DE technique is limited by its reduced reproducibility, issues in representing low abundant proteins, low molecular weight (LMW) and highly hydrophobic proteins, and difficulties in automating the process and accurately visualizing protein spots [79,81]. Hence, the 2-DE technique has been replaced by gel-free MS methods in plant research, including Fourier transform ion cyclotron resonance (FTICR), ion trap (IT), quadrupole (Q), time-of-fight (TOF), and orbitrap [82,83]. Q, TOF, and orbitrap are frequently used techniques in wheat proteomics (Table 1, Table 2, Table 3 and Table 4).

The development of ‘soft’ ionization MS techniques, such as matrix-assisted laser desorption/ionization (MALDI) and electrospray ionization (ESI) gave rise to the next generation of proteomics [82,84,85]. Despite challenges in protein identification from complex plant tissues, such as leaves and roots, and the requirement for a curated MS database [86], MALDI-TOF is a powerful tool with an improved protein extraction method [87,88]. Besides MALDI, shotgun proteomics is a popular proteomic strategy for identifying peptides from complex mixtures using hybrid MS, such as LC/MS and a database searching algorithm [89,90]. In shotgun proteomics, combining ESI and Q-TOF and/or linear ion trap TOF is popular for its efficiency in quantitative protein and PMTs identification [82]. 

With advances in MS techniques, genome sequencing data, and bioinformatic tools, label-based and label-free proteomics are popular gel-free shotgun methods [91]. Label-based sampling allows the samples to react with heavy or light versions of isotopes, such as isotope-coded affinity tags, stable isotope labeling using amino acids in cell culture, or isobaric tags, such as iTRAQ (isobaric tags for relative and absolute quantitation) [54]. Among all the techniques, iTRAQ is a popular method used in plant stress-responsive studies and trait improvement [81,92] (Table 1, Table 2, Table 3 and Table 4). In wheat, iTRAQ was first used in 2011, combined with 2DE-LC-MS/MS, to screen for drought stress-associated proteins [59] (Figure 2). However, iTRAQ is unsuitable for comparing large samples in a single run due to limited statistical efficiency and dependency on the availability of known peptide sequences [93]. Even though iTRAQ has been used in large-scale proteomic studies in plants, its accuracy is limited due to isotopic impurities and peptide co-fragmentation [94]. A relatively novel technique, tandem mass tag (TMT) is considered a highly sensitive proteomic platform for MS-based quantification of low abundance proteins [95]. Even though the TMT technique has been used successfully for stress-related proteomic studies in crops, resembling iTRAQ in terms of quantitative accuracy and precision, iTRAQ Q 4-plex identifies more peptides and proteins than TMT 6-plex [96]. On the other hand, LC-MS is popular for identifying proteins but requires a billion protein copies to classify an investigated protein. Long-read transcriptomics, cellular indexing of transcriptomes and epitopes by sequencing/spatial transcriptomics, and fluorescent fingerprinting methods have been proposed as LC-MS alternatives [97].

Recently label-free quantitative proteomics has gained popularity in stress related plant research as it is amenable to different types of biological samples and it constitutes a simple, reproducible, cheaper, more precise, and less error-prone alternative to stable isotope-based quantitative techniques [92,98,99]. Furthermore, unlike label-based techniques, label-free techniques can quantify all peptides in a sample allowing in-depth analysis of large-scale proteomics experiments because the estimation of protein abundances is based on measuring the intensity of peptide precursor ion for a given protein [100] or spectral counting [98]. The approach is performed using software, such as Proteome Discovers, Progenesis QIL, and MaxQuant [100]. However, due to the higher data dependency, higher variation in technical replication is common in label-free approaches [101]. The accuracy and precision of label-free quantitative data greatly depend on the resolution power of MS, higher scanning rates and exact chromatographic alignment [102]. To overcome the data dependency limitation, a new data-independent approach to sequential window acquisition of all theoretical fragment ion spectra mass spectrometry (SWATH-MS) has been used in rice, maize [103] and wheat [60] (Figure 1 and Figure 2).

## 4. Proteomics in Abiotic Stress Tolerance in Wheat

### 4.1. Heat Stress

Heat stress adversely impacts wheat growth, development, grain yield and grain quality [104]. It affects the different growth stages of wheat from seedling to grain filling; however, anthesis and early grain filling are the most sensitive stages [105]. Heat affects wheat yields significantly by affecting physio-biochemical processes, such as inhibiting photosynthesis through reducing the activity of RuBisCO activase and thus photochemical light-use efficiency. Altered photosynthesis and metabolic processes result in ROS production causing oxidation of cell membranes and proteins and DNA damage. ROS detoxification occurs with the help of antioxidant enzymes, such as superoxide dismutase (SOD), catalase (CAT), ascorbate peroxidase (APX), glutathione reductase and dehydroascorbate reductase (DHAR). Proteomic studies on heat stress in wheat revealed the involvement of proteins/enzymes related to the electron transport chain, glycolysis, protein synthesis and redox homeostasis in imparting heat stress tolerance [61,106,107,108]. Besides these, increased amounts of heat shock proteins (HSPs), suppressed anabolism and/or activated catabolism, sucrose synthase, glutathione S-transferase (GST) and anti-oxidant enzymes bestow heat stress tolerance.

Proteomics studies for heat stress tolerance in wheat began with the study of Majoul et al. [62], who investigated the effect of heat stress (at grain filling) on grain quality and dough properties using the 2-DE approach (Figure 2). The study reported that heat stress increased gliadins possibly due to heat stress elements in the upstream regions of gliadin genes, with glutenins being unaffected. As a result, the reduced ratio of glutenins to gliadins weakened the dough. The study revealed a significant decrease in one protein related to glucose-1-phosphate adenyltransferase, which plays a key role in starch synthesis, potentially reducing grain weight. Later, many proteomic studies were conducted on various wheat tissues at different growth stages (Table 1). Most of these studies were based on a post-anthesis heat stress treatment as it is the most sensitive stage and more likely to face heat stress in field conditions. Gupta et al. [63] carried out a proteomics study in wheat under heat stress at the seedling stage in ‘WH730’ (tolerant) and ‘Raj4014’ (sensitive) and derived recombinant inbred lines (RILs) to reveal the up-regulation of key proteins involved in photosynthesis (RuBisCO activase A and PEP carboxylase) and signal transduction (concanavalin A) in ‘WH730’ and tolerant RILs. The detailed findings of the studies undertaken post-anthesis are described below.

**Table 1:** Summary of proteomic studies on heat stress tolerance in wheat during the last decade (2015–2022).

Genotypes	Tissue and Developmental Stages	Treatments	Techniques	Effects	Genes/Enzymes	References
WH 730 (tolerant) and Raj 4014 (sensitive) with 10 extreme RILs	10-day-old seedlings (whole seedling sampled)	35 °C for 6 h	2-DE, MALDI-TOF/TOF -MS/MS	RuBisCO activase A, Con A and PEP carboxylase 1 were the key DEPs.	N/A	[63]
810 (tolerant) and 1039 (sensitive)	Flag leaf at 15 days post anthesis (DPA)	35 °C/26 °C (day/night) 5 days	2-DE, MALDI-TOF-MS	Proteins related to signal transduction, heat shock, photosynthesis, and antioxidants are upregulated, while those for nitrogen metabolism are downregulated.	N/A	[61]
Chinese Spring	Flag leaf (15 DPA)	37/17 °C (day/night) for 3 days	iTRAQ, LC-MS/MS	Chlorophyll synthesis, carbon fixation, protein turnover, and redox regulation were the most remarkable heat-responsive processes.	*GST* and *Trxs*	[109]
Jing411	Grains (sampled at 5, 10, 15, and 20 DPA)	40 °C for 2 h (12:00–14:00)	iTRAQ, LC -ESI Tandem MS/MS	256 DEPs for stimulus response, abiotic stress response, kinase activity and transferase activity.	*Calcineurin B-like*	[110]
Gaocheng 8901	Grains (sampled at 5, 10, 15, and 20 DPA)	40 °C for 2 h (12:00–14:00)	iTRAQ, LC-MS/MS	207 DEPs for energy metabolism, growth and development, and stress response were identified.	N/A	[111]
Triso	Flag leaf (10 DPA)	32 °C for 9 days and elevated CO_2_ (550 μmol/mol)	LC-MS/MS	Proteins for photosynthesis, antioxidant and protein synthesis pathways are downregulated.	*GST* and *Trx*	[112]
Chinese Spring	Grain in filling stage (15 DPA)	37 °C for 4 h	SDS–PAGE, TMT	A general decrease in protein synthesis components and metabolic proteins, but a significant increase in stress-response and storage proteins was found.	N/A	[113]
HD2985 (tolerant) and HD2329 (sensitive)	Pooled (Spikes, Stem Flag leaf) at pollination and grain filling stages	37 °C for 2 h	iTRAQ, LC-MS/MS	Carboxylase enzyme was the most abundant active enzyme under heat stress.	*HSP17*,* CDPK*,* Cu/Zn SOD*,* ADP glucophosphorylase and soluble starch synthase*	[114]
BWL4444	Mature grains	2 DPA to maturity; day heat stress (35/17 °C), Day–night heat stress (35/24 °C)	2DE, MALDI-TOF- MS/MS	Proteins related to the translation, gliadins, and low-molecular-weight glutenins are upregulated. Proteins related to glycolysis, photosynthesis, defense, and high-molecular-weight glutenins are downregulated.	*TaRSR1*,* OsbZIP58*,* glyceraldehyde-* *3-phosphate dehydrogenase*,* triose phosphate translocator* and *sucrose transporter*	[115]

N/A = not applicable; 2-DE = two- dimensional gel electrophoresis, MALDI = matrix assisted laser desorption/ionization, TOF = time-of-fight, MS = mass spectrometry, LC = liquid chromatography, ESI = electrospray ionization, iTRAQ = isobaric tags for relative and absolute quantitation, SDS–PAGE = sodium dodecyl sulfate–polyacrylamide gel electrophoresis, TMT = tandem mass tag, DEPs = Differentially expressed proteins.

Wang et al. [61] first conducted a proteomics study in flag leaf tissues in two wheat cultivars, ‘810’ (tolerant) and ‘1039’ (sensitive), exposed to heat stress during the grain filling stage. Differential expression (upregulation) of proteins related to photosynthesis, glycolysis, stress defense, heat shock and ATP production was observed. Under heat stress, HSP70 and RuBisCO activase increased in ‘810’ but decreased in ‘1039’; ‘810’ counteracted heat stress-induced ROS production by enhancing the expression of Cys peroxiredoxin BAS1. The proteins involved in energy transduction (CF1 subunits of ATP synthase) increased under heat stress, whereas nitrogen metabolism (glutamine synthetase) and the amino acid metabolism-related protein (aspartate aminotransferase) decreased in ‘810’, indicating reduced nitrogen and amino acid metabolism, respectively, under heat stress. Similarly, Lu et al. [109] identified 258 heat-responsive proteins in the flag leaves of wheat (Chinese Spring) under heat stress. In response to stress, upregulated heat-responsive proteins had significantly enriched protein folding, whereas downregulated heat-responsive proteins had enriched carbon fixation and translation indicating the negative impact of heat stress on photosynthesis and translation. The accumulation of GST, thioredoxin (Trxs), and HSPs under heat stress imparted redox homeostasis. Zhang et al. [111] analyzed the leaf proteome and revealed the up- and downregulation of 119 and 57 proteins, respectively, under heat stress, with most proteins related to photosynthesis, stress response and redox homeostasis. The maximum of the proteins was localized in chloroplasts, followed by cytosol and mitochondria. The reason for reduced grain weight may be attributed to the altered starch synthase activity (hampered starch synthesis). Kumar et al. [114] studied two wheat cultivars ‘HD2985’ (tolerant) and ‘HD2329’ (sensitive) and revealed the differential expression of 9425 heat stress-associated proteins (3600 up- and 5825 downregulated). A few unique proteins, such as Cu/Zn-SOD and γ-gliadin (seed storage protein), were upregulated in ‘HD2985’ and downregulated in HD2329. Furthermore, upregulation of HSP17, calcium dependent protein kinase (CDPK), Cu/Zn SOD and downregulation of AGPase, and soluble starch synthase was observed under heat stress. Heat stress altered the phenomenon of proton transport, photosynthesis, and ATP binding the most. Maximum heat stress-associated proteins were localized in chloroplasts, followed by mitochondria and ribosomes; thus, photosynthesis was most affected by heat stress. Signaling molecules (MAPKs and CDPKs), HSPs (HSP17, HSP20, HSP26, and HSP70) and antioxidant enzymes (SOD, CAT, and APX) imparted heat stress tolerance to organelles.

Zhang et al. [110] identified 256 differentially expressed proteins (DEPs) in grain tissues of ‘Jing411’ under heat stress. Most of the proteins were related to growth and energy metabolism, defense, and signal transduction. Heat stress mainly affected starch and sucrose metabolism and the protein synthesis pathway in the endoplasmic reticulum. Heat stress increased HSPs, protein disulfide isomerase (PDI), Myb-related proteins, 40S ribosomal protein, 60S ribosomal protein, and DnaJ protein, and decreased LMW glutenin subunits, adenosine diphosphoglucose pyrophosphorylase (ADPG-PPase), starch branching enzyme IIb (SBEIIb), pyrophosphate-fructose-6-phosphate 1-phosphotransferase (PFP), and sucrose synthase. Upregulation of PDI under heat stress helps with the proper folding of proteins, whereas Myb-related proteins, PFP, and 40S and 60S ribosomal proteins (RPs) help to maintain normal cell function. The upregulated HSPs (HSP90, HSP101, HSP26, HSP40, and mitochondrial HSP70) help in the correct folding and assembly of proteins. The protein–protein interaction network indicates the relative importance of coordinating proteins to form a functional protein network structure. CBL3, ERG3, BRI1, and DPBF4 formed an interaction network with multiple proteins. Calcineurin B-like (*CBL*) genes, which are key genes that regulate the calcium signaling pathway (stress signal transduction) were also identified. Zhang et al. [112] analyzed the kernel proteome in wheat cultivar ‘Gaocheng 8901′ and identified 207 DEPs under heat stress, which were mostly related to energy metabolism, growth and development, and stimulus and stress response. Among the identified DEPs, 78 governed KEGG signaling/metabolic pathways for protein synthesis in the endoplasmic reticulum, starch and sucrose metabolism, and the ribosome reaction. Wang et al. [113] carried out kernel proteomics at grain filling in the ‘Chinese Spring’ cultivar and reported 309 DEPs. Stress response and storage proteins increased, whereas protein synthesis components and metabolic proteins decreased. Heat stress tolerance was bestowed by higher energy investment into the stress response and starch deposition than metabolism and protein synthesis. The inhibition of protein synthesis (translation) under heat stress resulted from a reduction in aminoacyl tRNA synthetases. Recently, Chunduri et al. [115] investigated the effect of day and day–night combined heat stresses during grain filling and revealed the upregulation of proteins belonging to multiple pathways, including gliadins, LMW glutenins, and proteins involved in translation. In contrast, the high-molecular-weight glutenins and proteins involved in glycolysis, photosynthesis and defense were downregulated. Conclusively, heat stress-induced early gene expression increases/decreases in proteins belonging to multiple pathways to impart heat stress tolerance.

### 4.2. Salinity Stress

The seedling stage is considered the most sensitive stage to salinity stress and is a good indicator of adult stage tolerance to salinity. Hence, many proteomic studies on wheat have been conducted at the seedling stage under salinity stress (Table 2). Salinity stress at the seedling stage mainly affects photosynthesis, signal transduction, metabolism, osmotic homeostasis, energy production and transfer, and leaf antioxidant activities [116]. Gao et al. [64] first conducted proteomics in ‘Zhengmai 9023’ using a 2-DE approach and identified 83 differentially expressed spots. Subsequently, Q-TOF-MS analysis identified 52 salinity-responsive spots for transport-associated proteins, detoxifying enzymes, ATP synthase, carbon metabolism and protein folding. Some key proteins namely H^+^-ATPases, GST, ferritin and triosephosphate isomerase were upregulated under salinity stress. Kamal et al. [117] (Figure 2) first studied the proteome in leaf chloroplasts of ‘Keumgang’ and identified 65 unique proteins, mostly upregulated on the second and third days of stress (but downregulated on the first day of salinity stress). However, proteins related to ATP synthase and V-type proton ATPase subunits were downregulated during the entire salinity stress treatment. Under salinity stress, gradient upregulated proteins, such as cytochrome b6–f, germin-like-protein, the c-subunit of ATP synthase, glutamine synthetase, fructose-bisphosphate aldolase and S-adenosylmethionine synthase can be used as stress-responsive marker proteins. Jacoby et al. [65] conducted the first proteome analysis in the mitochondria of wheat shoot and root tissues under salinity. The enzyme, Mn-SOD exhibited differential abundance between varieties under salinity stress relative to the control. Glutamate dehydrogenase and aspartate aminotransferase were upregulated in shoots but downregulated in roots.

**Table 2:** Summary of proteomic studies on salinity stress tolerance in wheat during the last decade (2013–2022).

Genotypes	Tissue and Developmental Stages	Treatments	Techniques	Effects	Genes/Enzymes	References
Amphiploid developed from Chinese Spring and *Lophopyrum elongatum* (tolerant) and Chinese Spring (sensitive)	Mitochondria of shoots and roots, seedlings	200 mM NaCl	2D DIGE, MALDI-TOF/TOF MS	Manganese SOD (Mn SOD), serine hydroxymethyl transferase, aconitase, malate dehydrogenase, beta (β)-cyanoalanine synthase, glutamate dehydrogenase and aspartate aminotransferase were key DEPs.	N/A	[65]
Waha (*Triticum turgidum*) (tolerant)	Seed embryo and embryo surrounding tissues (germination stage)	NaCl (250 mM) for 42 h	LC-MS/MS Orbitrap Elite hybrid ion trap-Orbitrap MS	Methionine, auxin, metabolism, ROS managing and signaling imparted in salinity stress tolerance.	*S-adenosylmethionine synthetase*,* methionine methyltransferase*,* glutamate decarboxylase*,* 1-Cys peroxiredoxin* and *GST*	[118]
Roshan (tolerant) and Ghods (sensitive)	Leaves, 4-leaf stage seedlings	Hoagland solution with 200 mM NaCl	2DE, MALDI-TOF-TOF MS	RuBisCO activase, RuBisCO large and small subunits, chloroplastic trios-phosphate isomerase, cytosolic malate dehydrogenase upregulated.	N/A	[119]
T349 and T378 transgenic line with *GmDREB1* gene (maize promoter)	First expanded leaves, 10 days old seedlings	Kimura B nutrient solution with 300 mM NaCl	IEF gel, MALDI-TOF MS analysis	Osmotic- and oxidative stress-associated proteins, methionine synthase, glyceraldehyde-3-phosphate dehydrogenase, glutathione transferase, NADP-dependent malic enzyme and 2-cys peroxiredoxin BAS1 upregulated.	*GmDREB1*	[120]
Duilio (tolerant) (*T. turgidum*)	Leaf (5-days old seedlings)	Hydroponics-100 and 200 mM NaCl	LC-MS/MS	Plant defense, energy production and signal transduction related proteins are upregulated.	*CBSX3 (cystathionine β-synthase)* and *dehydrin*	[121]
*T. monococcum*	Leaves, seedlings	Hoagland solution with 80, 160, 240, and 320 mM NaCl	2DE, MALDI-TOF/TOF-MS	Cu/Zn SODs, GSTs, DHNs and LEA, 64 unique DAPs upregulated. Biomarkers for salinity stress response and defense: cp31BHv, betaine-aldehyde dehydrogenase, cytosolic (GS1), Cu/Zn SOD, MAT3, leucine aminopeptidase 2, and 2-Cys peroxiredoxin BAS1 were selected.	N/A	[122]
*Enterobacter cloacae* SBP-8 bacteria inoculated wheat cv. C-309	Whole plant, seedlings	Hoagland solution with 200 mM NaCl	LC-MS/MS	Cell wall (structure) strengthening proteins, such as tubulin, profilin, retinoblastoma, casparian strip membrane protein and xyloglucan endotransglycosylase, ion transporter (e.g., malate transporter), metabolic pathway and protein synthesis upregulated.	*Clp protease*,* Trxs h, cysperoxiredoxin, catalase* and *RuBisCO*	[123]
Han 12 (tolerant) and Jimai 19 (sensitive)	Roots, seedlings	Hoagland solution with 350 mM NaCl	iTRAQ, LC-MS, validation: RT-PCR, transgenic plant *Arabidopsis*	PPDK, LEA1 and LEA2 proteins imparted in salinity tolerance.	*TaPPDK*, *TaLEA1* and *TaLEA2*	[124]
Bobwhite	Roots and leaves, 2-week-old seedlings	Pots, 50 mM NaCl	LC-MS/MS, validation: qRT-PCR	Upregulated SODs, malate dehydrogenases, dehydrin proteins and V-ATPase protein, and Cu/Zn SODs, LEA and DHN proteins in roots and leaves, respectively.	*LEA* and *dehydrin*	[125]
Chinese Spring	Embryo proximal seed parts	Hoagland solution with 150 mM NaCl	Orbitrap Fusion Lumos LC-ESI MS/MS, validation: qRT-PCR	397 DAPs (133 upregulated/264 downregulated) were identified.	N/A	[126]
Qingmai 6 (tolerant)	Shoots and roots, 2-week-old seedlings	Water with 150 mM NaCl, and the same combined with 100 μM ethylene precursor ACC, and 150 μM ethylene signaling inhibitor 1-MCP	iTRAQ, Shotgun (Orbitrap Q Exactive HF-X MS)	DAPs: ribosomal proteins, nucleoside diphosphate kinases, transaldolases, β-glucosidases, and phosphoenolpyruvate carboxylases were upregulated; proteins related to metabolism played role in salinity response in wheat shoots.	*TaGSTU6*, *TaCCR*, *TaEXPB6*, *TaPOD*, *TaWRKY70* and *TaCYP450*	[16]
Chinese Spring	Seeds (endosperm)	Hoagland solution with 150 mM NaCl	Orbitrap Fusion Lumos LC-ESI MS/MS, validation: qRT-PCR	207 DEPs upregulated.	*TraesCS7B02G367600.1*, *TraesCS4A02G246100.1*, *TraesCS5D02G172800.1*, *TraesCS6A02G357200.1*, *TraesCS7A02G358200.1*, *TraesCS3A02G150800.1*, *TraesCS5A02G369900.1*, *TraesCS6A02G059800.1*, *TraesCS6A02G350500.1* and *TraesCS6A02G319300.1*	[127]
Zhongmai 175	Leaf chloroplast, seedlings	200 mM NaCl solution	Shotgun (Orbitrap Q Exactive HF-X MS), validation: qRT-PCR	Calvin cycle, amino acid metabolism, carbon and nitrogen metabolism, transcription and translation and antioxidation related 117 DAPs upregulated.	*Allene oxide synthase* *2-like*, *chaperone protein ClpC2*, *probable plastid-lipid-associated protein 2*, *phosphoglycerate* *kinase*, *phosphoglycolate phosphatase 1B*, *ribulose bisphosphate carboxylase large chain*, *50S ribosomal* *protein L2* and *sedoheptulose-1, 7-bisphos-* *phatase*	[128]
Scepter	Mature roots and root tips, Emergence of the second leaf (5 days-post transplant)	150 mM NaCl	Q-TOF, LC-MS	Translation related proteins, glycolytic enzymes, TCA cycle enzymes and ATP synthase subunits are downregulated.	*S-adenosylmethionine synthase, aspartate aminotransferase, O-methyltransferase, GST* and *phenylalanine ammonia lyase*	[26]

N/A = not applicable; 2-DE = two- dimensional gel electrophoresis, MS = mass spectrometry, Q = quadrupole, TOF = time-of-fight, SDS–PAGE = sodium dodecyl sulfate–polyacrylamide gel electrophoresis, 2D-DIGE= 2D difference gel electrophoresis, MALDI = matrix assisted laser desorption/ionization, LC = liquid chromatography, IEF = isoelectric focusing, iTRAQ = isobaric tags for relative and absolute quantitation, qRT-PCR = real-time quantitative reverse transcription PCR, ESI= electrospray ionization, DEPs = differentially expressed proteins, DAPs = differentially abundant proteins; SOD = superoxide dismutase, GSTs = glutathione S-transferases, DHN = dehydrin, LEA = late embryogenesis-abundant.

Capprioti et al. [121] imposed different levels of salinity stress (mild to high) and reported that the increased salinity levels mainly affected the proteins involved in energy production, signal transduction and plant defense (upregulated). Salinity stress affects photosynthesis through chloroplasts’ structure and composition (upregulation of chloroplastic UDP-sulfoquinovose synthase), energy production (reduced CO_2_ availability), and substrate availability. Unlike Caruso et al. [66], with the upregulation of ribulose-1,5-bisphosphate carboxylase oxygenase (RuBisCO), a small subunit was observed at 100 mM NaCl and higher concentrations. Chaperone proteins (help in proper folding and preventing misfolding of proteins), such as peptidylpropyl cis-trans isomerase and calnexin, (a calcium-binding protein), were upregulated under higher salinity [66]. Lv et al. [122] identified 81 spots corresponding to salinity stress and recovery and proteins mainly involved in regulatory, stress defense, protein folding/assembly/degradation, photosynthesis, carbohydrate metabolism, energy production and transportation, protein metabolism, and cell structure. Under the salinity stress, upregulation of GSTs, dehydrin (DHNs), and V-ATPase in roots, and late embryogenesis-abundant (LEA), and DHN in shoots, and Cu/Zn SODs in both tissues of wheat were found. Furthermore, salinity stress responsive biomarkers, such as betaine-aldehyde dehydrogenase, cp31BHv, Cu/Zn SOD, leucine aminopeptidase 2, cytosolic and 2-Cys peroxiredoxin BAS1 were identified through phosphoproteomics. In a subsequent salinity stress study, Han et al. [125] also found upregulated SODs, malate dehydrogenases and dehydrin proteins, V-ATPase protein in roots, and Cu/Zn, LEA protein, and DHN proteins in leaves. Maintaining cellular homeostasis in roots is an important strategy for salinity stress tolerance. Antioxidant enzymes, membrane intrinsic protein transporters, TFs, and ubiquitination related proteins are important for cellular homeostasis maintenance in roots. Pyruvate orthophosphate dikinase (PPDK) (encoded by *TaPPDK*) and LEA (encoded by *TaLEA1*, and *TaLEA2*) in wheat seedling roots also significantly contribute to salinity stress tolerance [124].

Improved antioxidative activities, accelerated protein synthesis, regulation of the transcription factors (TFs), and activated defense enzymes and lignin biosynthesis imparts salinity stress tolerance [123]. It was evident that during salinity stress in wheat, *Enterobacter cloacae* SBP-8 (a plant growth promoting bacteria) led to the upregulation of cell wall protecting proteins, such as casparian strip membrane protein, profilin, retinoblastoma, tubulin and xyloglucan endotransglycosylase, ion transporter proteins (e.g., malate transporter), defense proteins (e.g., Trxs), protease inhibitor and protein synthesis proteins, and the downregulation of lipid biosynthesis and protein degradation proteins [123]. The balance of the Na^+^/K^+^ ratio, uptake of Na^+^ in the shoot, and dissipation and specific energy fluxes and *OPAQUE1*, *NRAMP-2*, and transporter genes play important roles in salinity stress tolerance [129]. 

Ethylene-dependent salinity stress tolerance occurs through RPs activation (reduces ROS accumulation), chaperones synthesis, ROS scavenging, and altered carbohydrate metabolism; 1140 proteins and 73,401 genes were identified in a study, with significant differential expression of proteins including RPs, CDPKs, transaldolases, β-glucosidases, phosphoenolpyruvate carboxylases, SODs, and 6-phosphogluconate dehydrogenases. Among them, in root and shoot, eight DEPs including 48 RPs, and 49 DEPs were found, respectively [16]. 

In leaf chloroplasts of wheat seedlings grown under salinity stress, 117 upregulated DAPs were associated with the Calvin cycle, amino acid metabolism, antioxidation, carbon and nitrogen metabolism, transcription and translation. The upregulation of defense related proteins of chloroplasts, such as chloroplast ABC1-like family protein, type 2 phosphatidic acid phosphatase family protein takes part in salinity and drought stress tolerance [128]. Dissanayake et al. [26] recently carried out comparative profiling of root tip and mature root proteome under control and saline conditions. The study revealed the downregulation of translation related proteins, glycolytic and TCA cycle enzymes and ATP synthase subunits in root tips under salinity stress. Hence, salinity impaired protein synthesis capacity and energy production in root tips, but not in mature roots.

In durum wheat (*T. turgidum*), Fercha et al. [118] tested the potential of ascorbic acid treatment in imparting salinity tolerance. The study targeted the metabolic proteome in embryonic and embryo-surrounding tissues (separately) from germinating seeds, identifying 167 DEPs in embryos and 69 DEPs in embryo-surrounding tissues (since endosperm is a dead tissue). Of these DEPs, 129 proteins (45 upregulated and 84 downregulated) in embryos and 53 (26 upregulated and 27 downregulated) in embryo-surrounding tissues were differentially accumulated in an unprimed salinity-stressed condition. Most of the DEPs belonged to metabolism, energy, disease/defense, protein destination, and storage categories. Hence, salinity-induced reduced germination or dormancy results from an altered proteome in seeds. An ascorbate treatment can be used to break salinity-induced dormancy, helping germinating embryos survive early salinity stress [118]. Later, Yan et al. [126] carried out proteome profiling in germinating wheat seeds under salinity stress and identified 397 DEPs mainly related to small molecule metabolic processes, fatty acid degradation and the phenylpropanoid biosynthesis pathway. Similarly, Yan et al. [127] identified 207 DEPs in the endosperm of germinating seeds under salinity stress, mainly related to protein, amino acid and organic acid metabolic processes.

### 4.3. Drought Stress

Roots are the primary plant organ for combating the consequences of drought stress. Under moisture stress, roots struggle to supply sufficient water to aboveground plant parts, hampering photosynthesis due to osmotic stresses [130] that decrease NADP^+^ regeneration in the Calvin cycle [106,131], consequently disrupting the energy supply to plants. Cell function changes or damage under drought stress can be recovered by increasing the accumulation of amino acids and amines associated with osmoprotection and ROS scavengers, and proteins associated with glycolysis and glucogenesis. Amino acid biosynthesis proteins, such as glutamine synthetase also play an important role in improving drought stress tolerance through proline regulation [132]. Additionally, RuBisCO and abscisic acid (ABA) responsive proteins, such as GST, helicase, LEA, and proline play role in wheat drought stress tolerance [133]. To understand the molecular mechanism of drought stress tolerance of wheat more clearly, extensive proteomic studies have been carried out over the last decade (Table 3). 

The first study on drought proteomics combined with temperature stress under different nitrogen (N) concentrations, revealed that albumins-globulins and amphiphils (structural proteins) accumulated during early grain filling, and gliadins and glutenins (storage proteins) accumulated during early and late grain filling, respectively [67] (Figure 2). Under drought stress and with sufficient N supply, the accumulation of albumin and globulins increased by 60% with decreased temperatures [67]. Hajheidari et al. [134] reported the upregulation of ROS-associated proteins (GST and Trxs h), defense-associated proteins (α-amylase inhibitor), metabolism-related proteins (mitochondrial aldehyde dehydrogenase), and seed storage proteins (gliadin) in a drought stress-tolerant wheat genotype (Khazar-1) exposed to drought stress. Other studies have reported the role of gliadin in grain development and quality improvement under drought stress [135,136,137]. 

**Table 3:** Summary of proteomic studies on drought stress tolerance in wheat during the last decade (2013–2022).

Genotypes	Tissue and Developmental Stages	Treatments	Techniques	Effects	Genes/Enzymes	References
*Triticum turgidum* ssp. *Dicocoides*: TR39477 and TTD22, and *T. turgidum* ssp. *Durum*: Kızıltan	Leaf, seedling	No irrigation	2-DE, nano LC-ESI–MS/MS, qRT-PCR	Eleven drought stress-specific proteins (low peptide matches) were found. TMPIT1 (integral membrane protein) upregulated in wild emmer wheat.	*RuBisCO*, *MnSOD*, *GST* and *FNR*	[138]
Nesser (tolerant) and Opata (sensitive)	Roots, seedling	ABA (100 μM) or EtOH with growth media	iTRAQ, LC-MS/MS, qRT-PCR	Heat shock proteins (HSPs), O-methyltransferase and 2-caffeoyl CoA-methyltransferase upregulated in tolerant genotype.	*Rab24*, *dehydrin*, *fructose bisphosphate aldolase*, *lipoxygenase 1* and *2*, *calnexin*, *elicitor responsive protein 3-like* and *caffeic acid o-methyl transferase*	[68]
Yannong 19	Leaves, reproductive	Drought (35–60% relative water content in soil)	2-DE, MALDI-TOF/TOF-MS	Photosynthesis and carbon metabolism associated proteins reduced yield under severe drought combined with low temperature.	*Cu/Zn SOD, tAPX*, *MnSOD* and *CAT*	[139]
Hanxuan 10 (tolerant) and Ningchun 47 (sensitive)	Leaf, seedling	20% PEG-6000 in Hoagland solution	TiO_2_, label free LC-MS/MS	Sensors related to Ca^2+^ showed differential expression at phosphorylation. Phosphorylated proteins (H+-ATPase, MSSP2, PP2C, TaABI5, WCOR615 and WAL17) are upregulated to improve drought stress tolerance.	*TaABI5-1*, *MYB1R1* and *bHLH*	[140]
Gaocheng 8901, Jagger and Nongda 3406	Seed, reproductive	7–12% soil content	SDS-PAGE, MALDI-TOF/TOF-MS	Albumin and gliadin upregulated significantly.	N/A	[135]
KTC86211	Leaf, seedling	PEG- 6000 (−0.50 Mpa) spray	2-DE, MALDI-TOF/TOF-MS	ROS scavenging proteins (ascorbate peroxidase, GST, thiol-specific antioxidant protein) primarily upregulated.	N/A	[141]
SERI M 82 (tolerant) and SW89.5193/kAu2 (sensitive)	Leaf and root, seedling	20% field capacity	2-DE, nanoLC-MS/MS, qRT-PCR	Cell biogenesis and degradation-related proteins significantly upregulated in leaf and root of tolerant genotype.	*Ascorbate peroxidase*, *ATP synthase subunit β*, *GST* and *16.9 kDA HSPs*	[142]
Hanxuan 10 (tolerant) and Chinese Spring (sensitive)	Roots, leaf, and intermediate sections (IS) between roots and leaf (IS), seedling	20% PEG-6000 in ½ Hoagland solution, drought recovery	2-DE, MALDI-TOF/TOF-MS	A higher percentage of proteins upregulated in roots than in leaves and IS during drought stress but downregulated during recovery. HSPs significantly upregulated in all organs.	N/A	[130]
Erebuni (*T. boeoticum*)	Leaf and root	20% PEG-6000 in 1/2 Hoagland solution	2-DE, MALDI-TOF/TOF-MS	Abscisic acid increased higher in leaves than roots to improve drought stress tolerance. Signal transduction proteins, and UDP-glucose/GDP mannose dehydrogenase, ribulose-phosphate 3-epimerase, transketolase and transaldolase-like protein are upregulated, but proteins related to protein metabolism and glycolysis are downregulated in roots.	N/A	[143]
Transgenic wheat lines (08 T(1)-27 and 08 T(1)-47) containing maize phosphoenolpyruvate carboxylase (*PEPC*) gene developed from Zhoumai19	Leaf and root, reproductive	30–35% soil moisture	2-DE, MALDI-TOF-MS	ATP synthesis subunits, ferredoxin-NADP reductase and S-adenosylmethionine, chloroplast glyceraldehyde-3-phosphate dehydrogenase, chlorophyll A-B binding protein and phosphate diakinase upregulated in transgenic wheat.	*PEPC*	[144]
Xihan No. 2 (tolerant) and Longchun 23 (sensitive)	Leaf and root, seedling	30% moisture content	2-DE, MALDI-TOF/TOF-MS	Proteins associated with photosynthesis, stress defense and detoxification played the most important role in yield improvement during drought stress.	N/A	[145]
Kundan (tolerant) and Lok1 (sensitive)	Leaf, seedling and reproductive	50% and 75% relative water content in leaf and rehydration for recovery	2-DE, MALDI-TOF-MS, western blotting	Proteins related to carbon metabolism, amino acid, defense and antioxidation took part in drought stress tolerance.	N/A	[146]
Yumai34	Leaf, seedling	0.05 mM NaHS and PEG 6000 in Hogland solution	SDS-PAGE, iTRAQ, nano-LC-MS/MS, RT-PCR	Carbon metabolism and protein synthesis associated proteins increased, and photosynthesis and signal transduction proteins downregulated in PEG with NaHS treated genotypes.	Genes associated with W5A5Z6, W5A2Y8, W5BBW7, W5IAG4, W5F3S8, W5EDB0, W5H6J0, W5BQ07, and C1K737 proteins	[147]
Shaanhe 6 (tolerant) and Zhengyin 1 (sensitive)	Leaf, seedling	70%, 50%, 40%, 30%, and 20% field capacity	SDS-PAGE, LC-MS/MS, western blotting	LEA protein helped in drought stress tolerance.	*lea* genes	[148]
Kavir (tolerant) and Bahar (sensitive)	Leaf, seedling	No irrigation for a week	2D-PAGE, LC-MS/MS	ADP-glucose pyrophosphatase, GST, glyoxalase enzymes and phosphoribulokinase downregulated in the sensitive genotype, and soluble inorganic pyrophosphatase is downregulated in both genotypes.	N/A	[149]
Zhongmai 175	Flag leaf and grain, reproductive	No irrigation	2D-DIGE, MALDI-TOF/TOF-MS, western blotting, qRT-PCR	Proteins associated with photosynthesis and energy metabolism, and carbon metabolism and stress found in flag leaves and grain, respectively, responded during drought stress.	N/A	[69]
Jinmai 47	Leaf, seedling	20% PEG-6000 in 1/2 Hoagland solution	iTRAQ, LC/MS, qRT-PCR	Citrate synthase, pyruvate dehydrogenase E1 component subunit alpha and aconitate hydratase upregulated during drought stress. Redox regulating proteins, chaperone proteins and enzymes proline biosynthesis are also upregulated, but RuBisCO activase small subunit downregulated.	N/A	[150]
Yan995	Leaf, seedling	25% PEG-6000 in 1/2 Hoagland solution and 40% field capacity	iTRAQ, MS/MS, qRT-PCR	Formate tetrahydrofolate ligase, glyceraldehyde-3-phosphate dehydrogenase, malate dehydrogenase 2, phosphoglycerate kinase, RuBisCO, and serine hydroxymethyl-transferase significantly downregulated in both type stresses. Amino acid synthesis associated proteins hampered plant growth during stress.	Genes associated with W5E659, W5EN32, W5ATV6, W5BAB9, W5ETI9, G8D5C5, W5DTC2 and W5FL86 proteins	[151]
Arg (tolerant) and Arta (sensitive) and F_6_ lines of their cross	Leaf, seedling	No irrigation	Linear ion trap mass spectrometer	Photosynthesis and stress-associated proteins downregulated and upregulated, respectively. Proline and malondialdehyde played a significant role in drought stress improvement.	N/A	[152]
Chinese Spring	Seed, reproductive	20% PEG 6000 in modified 1/4 Hoagland solution	Label-free nano LC-MS/MS	4-coumarate-CoA ligase, shikimate O-hydroxycinnamoyl transferase, caffeic acid O-methyltransferase, caffeoyl CoA O-methyltransferase, cinnamyl-alcohol dehydrogenase, and peroxidases downregulated.	N/A	[126]
Arg (tolerant) and Moghan3 (sensitive)	Leaf, reproductive	No irrigation after pollination to harvest	2-DE, MALDI TOF/TOF-MS	Proteins associated with photosynthesis, stress defense and detoxification played the most important role in higher yield during stress.	N/A	[153]
PAN3478	Seed, reproductive	No irrigation	2-DE, LC–MS/MS	α-gliadin upregulated. High molecular weight glutenin proteins expressed differentially for wheat quality.	N/A	[136]
Yangmai 16	Root apex, seedling	Drought priming by 5% (−0.37 MPa) and 15% (−0.78 MPa) PEG in Hoagland solution	iTRAQ, MS/MS	Phytohormones (auxin, cytokinin, brassinosteroids, ethylene, abscisic acid, jasmonic acid and salicylic acid) downregulated during drought stress.	N/A	[51]
TRI 5630 (tolerant) and White Fife (sensitive)	roots, leaves and seeds, reproductive	71.11% field capacity	SDS-PAGE, LC−MS/MS	3-ketoacyl-CoA synthase and ATP-binding cassette transporter regulated cuticular wax biosynthesis in wheat leaf and improved drought stress tolerance.	N/A	[154]

N/A = not applicable; 2-DE = two- dimensional gel electrophoresis, MS = mass spectrometry, Q = quadrupole, TOF = time-of-fight, SDS–PAGE = sodium dodecyl sulfate–polyacrylamide gel electrophoresis, 2D-DIGE = 2D difference gel electrophoresis, MALDI = matrix assisted laser desorption/ionization, LC = liquid chromatography, iTRAQ = isobaric tags for relative and absolute quantitation, qRT-PCR = real-time quantitative reverse transcription PCR, ESI = electrospray ionization.

Leaf proteomics identified that flag leaves are more drought sensitive than grain as drought stress significantly affected photosynthesis-associated proteins localized in flag leaf chloroplasts [69]. In contrast, drought stress significantly affected carbon metabolism and stress-associated proteins in grain. Under drought stress, L-ascorbate peroxidase-1 was significantly upregulated in grain and improved stress tolerance through maintaining the balance in the ascorbate–glutathione cycle and improving H_2_O_2_ removal efficiency [69]. Proteins associated with the TCA cycle (citrate synthase, pyruvate dehydrogenase E1 component subunit alpha and aconitate hydratase), redox regulation LEA proteins (Rab protein), chaperon proteins, amino acid metabolism or proline biosynthesis (delta-1-pyrroline-5-carboxylate synthase, ornithine aminotransferase), and carbohydrate metabolism proteins (sucrose synthase 4) were upregulated. However, the photosynthesis-related protein RuBisCO activase small subunit was downregulated in leaves of ‘Jinmai 47’ at drought stress [150]. PEG-induced drought and soil drought stress significantly led to the upregulation of other carbohydrate metabolism proteins, such as pyrroline-5-carboxylate dehydrogenase and pyrroline-5-carboxylate synthetase, and significant downregulation of phosphoglycerate kinase, formate tetrahydrofolate ligase, phosphoglycerate kinase, glyceraldehyde-3-phosphate dehydrogenase and serine hydroxymethyl-transferase in wheat leaves. In addition, drought stress affected the metabolism of glutathione, ascorbate, lignin, starch, sucrose, amino acid, proline, and polyamine metabolism in wheat leaves [151]. To combat drought stress and increase yield, besides proteins associated with photosynthesis (fructose-bisphosphate aldolase), stress defense (HPs) and detoxification (superoxide dismutase, peroxidase), transporter proteins (ATP-binding cassette), and protein synthesis proteins (60S ribosomal protein L31-1) also play an important role [153].

Kang et al. [155] reported that salicylic acid (SA; 0.5 mM) enhanced the expression of drought tolerance proteins, viz. carbohydrate metabolism associated proteins, such as isocitrate dehydrogenases, malate dehydrogenase (MD), 6-phosphogluconate dehydrogenase, and triosephosphate isomerase, and those involved in glutamine production (glutamine synthase) [155]. SA, an agent of stress hormone networks in plants [156] also activates plant defense responses under drought stress through Trxs based redox regulation [146]. Photosynthesis-related proteins (fructose 1,6-bisphosphatase and ATPase) play a significant role in drought stress tolerance. However, other photosynthetic proteins, such as RuBisCO, amino acid metabolism associated proteins, other redox signaling and defense related proteins (GST) including Trxs were upregulated in tolerant genotypes during drought stress recovery. Increased seed number and weight were evidence of drought stress tolerance in SA treated wheat [146].

In an integrative proteomics study on roots, leaves, and intermediate sections between roots and leaves (IS), most DEPs in roots were upregulated during stress but downregulated during the recovery, and roots/IS shared more DEPs than leaves/IS [130]. Under stress, most DEPs were associated with defense and carbon metabolism in roots, and photosynthesis in leaves, while those related to IS were associated with both. In IS of ‘Hanxuan 10’ (tolerant), calreticulin-like protein (signal transduction protein) was upregulated during stress but downregulated during recovery. Upregulation of HSPs during drought stress—HSP60, HSP70, and HOP (Hsp70-Hsp90 organizing protein) in roots and HSP104 and HSP70 in leaves, and 23.5 kDa HSP in both roots and leaves indicated increased phosphorylation during the stress. Peroxidases (defense-related proteins) were upregulated significantly in roots and IS during drought stress. Proteins, such as the RuBisCO large subunit, oxygen-evolving enhancer protein 2 (OEE2), and Ribulose-1,5-bisphosphate carboxylase/oxygenase activase associated with photosynthesis were upregulated in the tolerant genotype during drought stress. However, except for OEE2, all were downregulated during the recovery [130]. Dehydroascorbate reductase, associated with the photosynthesis, transpiration and antioxidant activity of catalase [157], was upregulated in leaves and IS during drought stress [130]. The role of HSPs in drought stress tolerance was also reported in the first comprehensive root proteome study [68]. In a comparative leaf and root proteomics study, significant upregulation and downregulation of glucan endo-1,3-β-glucosidase, MD, peroxidase, and S-adenosylmethionine synthase in roots and leaves of tolerant and sensitive genotypes, respectively, were found during drought stress. However, S-adenosylmethionine synthase, ferredoxin-NADP(H) oxidoreductase, and hairpin binding protein 1 were upregulated in leaves, while germin-like proteins were significantly upregulated in the roots of the tolerant genotype [142]. In a leaf, root, and seed proteomics study under drought stress, it was revealed that the leaf and root proteomes varied less than the seed proteomes. Interestingly, the authors identified 3-ketoacyl-CoA synthase and ATP-binding cassette transporter enzymes in leaf tissue which function in drought avoidance through circular wax biosynthesis [154]. A recent study suggested maintaining RuBP synthesis, and controlling starch biosynthesis through the overexpression of ADP-glucose pyrophosphatase, increasing the glutathione response, accumulating organic osmolytes, and downregulating auxin production to develop drought stress-tolerant wheat using omics-assisted breeding [149]. 

The above studies demonstrate that drought stress tolerance proteins are often organ-specific, and root proteins alter more during stress than other tissues, such as leaf and seed. Commonly, drought stress affects photosynthesis, carbon metabolism, proline, redox scavenging, defense, or detoxification-related proteins in wheat.

## 5. Proteomic Approaches to Study RSA

Plants trigger physiological, molecular and biochemical modifications in response to stress [158] affecting the growth and development of RSA to combat the stress [13,27,159]. Hence, the exploration of the molecular mechanism of RSA alteration for stress tolerance is important for developing stress-tolerant elite wheat varieties. Proteomics is an outstanding tool for analyzing quantitative trait (e.g., RSA) modifications at the protein level [23]. Targeted root proteins associated with different stress responses, such as heat, and Al stress tolerance were studied in the 1980s and 1990s using 2-DE [160,161,162], but the first proteomic study on total root tissue was reported in 2005 [70] (Figure 2). Using 1-DE and 2-DE, Bahrman et al. [70] found that ‘Récital’ seedlings had higher fresh root weights and nitrate levels under nitrogen stress than ‘Arche’ seedlings due to overexpression of 23 DEPs. However, with advances in high-throughput analyses and developed MS facilities, root proteomics shifted from selected protein spots to protein quantification. This section covers the recent progress of RSA proteomics in wheat using modern analytical techniques (Table 4).

**Table 4:** Summary of proteomics studies on wheat root traits of root system architechture (RSA) during the last decade (2013–2022).

Root Traits	Genotypes	Tissues	Techniques	Important Findings	Genes/Enzymes	Validation	Treatments	References
Dry mass	Yumai 34	Total root	2-DE, tandem MS	Higher lipid peroxidation by malondialdehyde (MDA) at roots caused more sensitivity of roots than leaves under copper (Cu) toxicity. Upregulated glutathione S-transferase (GST) and downregulated MDA led to improved Cu stress tolerance.	*GST*	Quantitative real-time PCR (qPCR)	Cu stress (100 µM CuSO_4_·5H_2_O)	[163]
N/A	Opata and Nesser	Total root	iTRAQ, LC-MS/MS	Heat shock proteins, signal transduction pathway, secondary metabolism, and lignin metabolism associated proteins helped to drought stress tolerance through improved root growth.	*Rab24*	qPCR	Drought (ABA (100 μM) or EtOH with growth media)	[68]
N/A	Keumgang	Mitochondria from root	Tricine SDS-PAGE, LTQ–FTICR MS	Proteins associated with translation, energy metabolism and amino acid synthesis were important to supply energy for root growth.	N/A	N/A	Controlled (soil in a greenhouse)	[164,165]
Depth	F_2_ generation from QTL isolines 178A and 178B, and 10 commercial varieties	Total root	2D-DIGE	Primary rooting depth was reduced due to accumulation of oxygen in root tip and size of meristem and inhibition of peroxidases (PODs) activity, brassinosteroid (BR) by *TaTRIP1*. 24-epibrassinolide increased root meristem size.	*TaTRIP1* and *POD*	qPCR and western blot	Controlled (hydroponic)	[166]
Depth, fresh mass	Keumkang	Total root	2-DE gel, nano-LC/MS	Root elongation is reduced with high Aluminum (Al) concentration due to upregulation and downregulation of 19 and 28 proteins, respectively.	N/A	N/A	Al stress (0, 100 and 150 µM AlCl_3_)	[23]
Depth	Yumai 34	Total root and leaf	iTRAQ, LC-MS/MS	117 differential expressed proteins (DAPs) were found in wheat root under mercury (Hg) stress. Upregulated ADP-ribosylation factor GTPase- activating protein and antioxidant enzymes regulated root growth under Hg stress.	N/A	N/A	Heavy metal (different concentrations of HgCl_2_ with Hoagland solution)	[167]
Dry mass	Transgenic wheat containing Phosphoenolpyruvate carboxylase (*PEPC*) gene of maize developed from wheat variety Zhoumai19	Leaf	2-DE gel, MALDI-TOF-MS	Prostatic acid phosphatase fibrillin and protein related to methionine synthesis increased root growth and root mass due to the influence of PEPC, and so, improved drought stress tolerance.	*PEPC*	N/A	Drought (30–35% relative soil water)	[144]
Depth, fresh mass	Jiami 19 (sensitive) and Han 12 (tolerant)	Total root	iTRAQ, nano LC-MS/MS	Pyruvate, phosphate dikinase, late embryogenesis abundant (LEA) protein1 and LEA2 proteins increased rooting depth and fresh root mass and thereby improved salinity stress tolerance.	*TaPPDK1*,* TaLEA1* and *TaLEA2*	RT PCR, transgenic *Arabidopsis* and soybean	Salinity (0.4% soil salinity, 150 and 200 mM NaCl)	[133]
Depth and volume	Seri M82 (sensitive) and CIGM90.863 (tolerant)	Total root	TMT, LC-MS/MS	Upregulated proteins related to anaerobic adaptation and fermentation, such as alcohol dehydrogenases might increase root volume to improve waterlogging tolerance.	*TaBWPR-1.2#2 *and* TaBWPR-1.2#13*,* Mn-SOD* and *NADK3*	qPCR	Waterlogging (hypoxic by N_2_ gas bubbling and 2.0 mg/L O_2_ in water)	[50]
Depth	XY54 and J411	Total root	iTRAQ, LC-ESI-MS/MS	Eighty differentially expressed proteins (DEPs) associated with the steroid biosynthesis pathway, and peroxidases controlled rooting depth (primary rooting depth, and total rooting depth). Brassinosteroid biosynthesis pathway mediated ROS distribution contributed to long primary root growth through determining root meristem size.	Peroxidases related genes	qPCR	Controlled(greenhouse)	[168]
Depth and dry mass	Yumai 34	Total root and leaf	iTRAQ, LC-MS/MS	Eight-hundred and sixty-six nitrogen (N_2_) deficiency associated proteins were found in the root. Wheat seedlings with silenced zeaxanthin epoxidase had reduced dry mass and high sensitivity to stress.	N/A	N/A	N_2_ stress (N_2_ free Hoagland solution)	[169]
Depth	RIL from XY54 × J411	Total root and leaf	iTRAQ, LC-ESI-MS/MS	Lower N_2_ promotes longer root growth; 84 DAPs increased root growth. Four and one of glutathione metabolism related DAPs were upregulated and downregulated, respectively, and associated with longer root growth under lower N_2_.	N/A	qPCR	N_2_ stress	[170]
N/A	M1019 (tolerant) and Xinong20 (sensitive)	Total root	TMT, LC-MS/MS	Tolerant genotype had higher cadmium (Cd) in root cell walls than cell fluid and cytoplasm. Upregulation of DEPs associated with transferase activity, transferring glycosyl groups and metal iron binding helped in Cd stress tolerance.	N/A	N/A	Cd (CdCl_2_ stress)	[171]
N/A	HD2985 (tolerant) and HD2329 (sensitive)	Leaf, stem, and spike	iTRAQ, LC-MS/MS	HSP17 and HSP70, calcium-dependent protein kinase (CDPK) and Cu/Zn SOD, and defense associated proteins were upregulated in roots which might improve heat stress tolerance.	*β-actin*,* HSP70*,* HSP17*,* CDPK*,* Cu/Zn SOD*,* f*,* Rca*,* OEEP*,* SucSyn*,* AGPase*,* SSS*,* SBE*, and *α-amylase*	qPCR and immunoblotting	Heat stress	[114]
N/A	Qingmai 6	Total root and leaf	iTRAQ, LC-MS/MS	Sixteen and three DAPs were found in roots at ethylene precursor ACC and ethylene inhibitor treatment, respectively. Ethylene dependent salinity response in root changed significantly due to the accumulation of 48 ribosomal proteins.	*LOXs*,* UDPGs*,* GLUDs*,* PALs*,* 6-PGDHs*,* GSTs*,* BGLUs*,* PODs*, and *OXOs*	qPCR	Salinity stress (ethylene dependent salinity stress)	[16]
Depth	TRI 5630 (tolerant) and White Fife (sensitive)	Total root	SDS-PAGE, LC-MS/MS	The rooting depth of both genotypes increased under drought stress might be due to the upregulation of β-glucosidase.			Drought (71.11% field capacity)	[154]
Total length, number, average diameter, dry mass and specific length	Jimai 22	Total root	SDS-PAGE, LC-MS/MS	Total root length and specific root length decreased significantly due to upregulated peroxidase enzyme and phenylalanine ammonia-lyase. Proteins related to GST and phenylpropanoid biosynthesis upregulated and played an important role in root development and oxidative stress tolerance.	*A0A3B6K9P2*,* Q8RW0*,* A0A3B6JL78*,* TraesCS3D02G344800*, and *TraesCS3A02G350800*	N/A	NH^4+^/NO^3−^ ratios	[171]
N/A	Wyalkatchem	Root tip and root mucilage	Q-TOF/LC-MS	Root mucilage proteins, such as endopeptidase and oxidoreductase or carbohydrate binding played role in root development. Cell wall modified and defense mechanism influenced by P-starvation induced proteins, peroxidase, protease and chitinase localized at the root tip apoplast.	N/A	Multiple rection monitoring	Phosphorus starvation (250 μM KH_2_PO_4_ for 10 days)	[25]
Total length and dry mass	Scepter	Total root	Q-TOF/LC-MS	Root tip growth reduced more than mature root under salinity stress due to decreased abundance of TCA cycle enzymes, such as aconitate hydratase, and ATP synthase subunits, such as subunit β.	*TraesCS5A01G505000.2* and *TaesCS1A01G379000.1*	N/A	Salinity (150 mM NaCl)	[26]

N/A = not applicable; 2-DE = two- dimensional gel electrophoresis, LC = liquid chromatography, MS = mass spectrometry, iTRAQ = isobaric tags for relative and absolute quantitation, LTQ–FTICR = linear trap quadrupole- fourier transform ion cyclotron resonance, SDS–PAGE = sodium dodecyl sulfate–polyacrylamide gel electrophoresis, MALDI = matrix assisted laser desorption/ionization, TOF = time-of-fight, ESI = electrospray ionization, TMT = tandem mass tag, Q = quadrupole, qRT-PCR = real-time quantitative reverse transcription PCR, 2D-DIGE = 2D difference gel electrophoresis.

Uncovering the wheat genome sequence [172] opened new avenues for exploring root proteomics. Alvarez et al. [68] first reported the comprehensive root proteome of ‘Nesser’ (drought tolerant) and ‘Opata’ (drought-sensitive) seedlings under drought stress using iTRAQ and LC-MS/MS (Figure 2). Among 805 ABA (cause of drought stress) responsive proteins in roots, six LEA proteins, protein phosphatase and an ABA-responsive protein caused drought sensitivity. ‘Nesser’ had more HSPs (HSP90 and HSP70) and other proteins associated with the signal transduction pathway, such as phosphatases, calcium-dependent and mitogen-activated protein kinases, multiple GTP-binding proteins, vacuolar ATPase, and proteins associated with cell wall biogenesis than ‘Opata’, indicating the role of these proteins in imparting drought stress tolerance. Cell wall biogenesis proteins play a role in lignin metabolism (cell wall structure) in roots [68] and are hence important for root growth and development. Similarly, Liu et al. [143] used a comparative proteomics approach in wild wheat (*T. boeoticum*) to identify 80 unique root proteins and six common roots and shoot proteins under drought stress. Glutamate decarboxylase, proteasome subunit β type-7-A, and HSP 70 were upregulated under drought stress; after 48 h of drought stress, malondialdehyde (MDA) increased by 23.33%. In a recent study, increased rooting depth of ‘White Fife’ (sensitive) and ‘TRI 5630’ (tolerant) was found at the developmental stage (heading emergence) during drought stress. Proteins, such as β-glycosidase, β-amylase, peroxidase, proteins of eukaryotic translation initiation factor 3 subunit, and protoporphyrinogen oxidase were associated with increased root length. Under drought stress, β-glycosidase and β-amylase are important for carbohydrate metabolism activation in roots; the former enzyme also improves root growth and development via cell wall modification and cellulose hydrolysis [154].

Heat stress affected RSA, especially total root length more than shoot growth [173]. Heat shock proteins, such as HSP17 and HSP70, and signaling molecules of stress associated active proteins, such as calcium-dependent protein kinase, and antioxidant enzymes, such as Cu/Zn-SOD were upregulated in the root of the tolerant variety [114].

Heavy metals affect protein expression in roots. Cadmium (Cd) stress tolerance improved due to Cd accumulation in root cell walls. Genotype ‘M1019’ had improved Cd tolerance through the upregulation of 268 proteins associated with carbon fixation via glutathione transferase activity, transferring glycosyl groups and metal-binding activities relative to ‘Xinong20’ [174]. Under aluminum (Al) stress, 19 proteins, including S-adenosylmethionine synthetase, β-amylase, MD, UDP-D-glucuronate decarboxylase, and ascorbate peroxidase were upregulated in roots [23]. Besides stress tolerance improvement, root proteins play an important role in nutrient mobilization for ease of uptake. Recently, Staudinger et al. [25] reported the role of root mucilage proteins in mobilizing phosphorus (P). Under P starvation, 2287 and 333 protein groups were identified in root tips and root mucilage, respectively, with 186 unique proteins in root mucilage, of which endopeptidase and oxidoreductase were considered to play an important role in root development. Apoplastic localized P-starvation induced proteins (peroxidase, protease, and chitinase) that regulate cell wall modifications and defense mechanisms were upregulated in the root tip and mucilage (relatively higher in mucilage), indicating their role in root development and stress tolerance. However, the first cell wall proteomics of wheat (Figure 2) revealed that upregulation of chitinase under flooding stress helped in flooding tolerance; however, the downregulation of β-glucanase, β-glucosidase, methionine synthase, and glyoxalase inhibited cell wall elongation in wheat seedling roots, significantly reducing the rooting depth and fresh mass [71]. A recent study found that lignin biosynthesis (peroxidase enzyme and phenylalanine ammonia-lyase) played an important role in reducing total root length and specific root length under high NH_4_^+^/NO_3_^−^ ratios [171]. However, lignification helps to protect the roots from oxidative stress due to ROS by restricting excess nitrogen supply. Roots were also protected by upregulating GST and phenylpropanoid biosynthesis pathway associated proteins, which played an important role in root development [171].

Salinity affects the root directly, altering the root proteome composition. Thirty-four upregulated and 16 downregulated salinity stress-responsive proteins were identified in ‘Jiami 19’ (sensitive) and ‘Han 12’ (tolerant) seedlings [124]. Three salinity tolerance genes (*TaPPDK1*, *TaLEA1*, and *TaLEA2*) encoding pyruvate phosphate dikinase, LEA protein1, and LEA2, respectively, were validated in transgenic *Arabidopsis* and soybean root hairs. Under saline conditions rooting depth and root fresh mass significantly increased in transgenic genotypes relative to the wildtype and consequently had higher salinity tolerance. Root proteins, such as ubiquitin-like protein, speckle-type POZ protein, F-box proteins, and coronatine insensitive 1 also play an important role in salinity stress tolerance [124]. In another study, 80 primary root growth (PRG)-associated DEPs were identified in roots of ‘XY54’ (32 proteins upregulated and 48 downregulated) which caused longer primary roots than ‘J411’. Class III peroxidases are important proteins for long primary root growth through increasing the brassinosteroid biosynthesis pathway and mediating ROS distribution. Peroxidases also play an important role in determining meristem size in root tips [168]. Xu et al. [170] identified 84 DAPs for wheat PRG at low N, of which glutathione transferases, aminopeptidase, and glutathione peroxidase increased, and L-ascorbate peroxidase 1 decreased. Zinc finger and amino acid transporters and cinnamoyl-CoA reductase were reported as putative proteins for root dry matter and root diameter, respectively [175]. Recently, Dissanayake et al. [26] found that the total root length and root diameter (average) of ‘Scepter’ significantly decreased under salinity stress. Using targeted and untargeted proteomics, 50 and 172 DAPs were identified in mature roots and root tips, respectively, under three and six days of stress. Alcohol dehydrogenase, phenylalanine ammonia-lyase, and O-methyltransferase, which produce secondary metabolites, redox reaction-associated proteins (e.g., GST and peroxidase), and a stress-associated protein (endo-1,3(4)-β-glucanase 1) were upregulated in root tips under stress. In contrast, secondary metabolite-associated proteins and peroxidases were upregulated in mature roots. However, most of the proteins in both tissues were associated with protein synthesis and degradation function. The decreased abundance of enzymes related to the TCA cycle (e.g., aconitate hydratase) and ATP synthase subunits (e.g., subunit β) hampered root tip growth more than mature roots under salinity stress, indicating that root tips are more sensitive to salinity stress than mature roots. 

Despite unveiling the role of the wheat root proteome in root growth and development under different environmental scenarios and control conditions, the total proteins of root tissue remain unexplored due to their complex polyploid genomic construct. Additionally, the complete protein database of wheat roots is yet unavailable, and thus, the use of protein markers for root improvement is limited [176]; further exploration of functional proteins in wheat roots is needed to improve stress tolerance and ultimately protein yield.

## 6. Limitations and Potential of Proteomics for Abiotic Stress Tolerance and RSA in Wheat

Plant abiotic stress responses include the expression of stress-responsive proteins; proteomics is an excellent tool for identifying those proteins. Compared to other model crops, such as *Arabidopsis* and rice, comparative proteomic studies in wheat are limited [38,69] due to the high cost and complex nature of proteomics and the polyploid wheat genome. Furthermore, the proteomic databases of other crops, such as rice [177], maize, and the model plant *Arabidopsis* [178] are available, but the wheat proteomic database [179] is incomplete. Such limitations make it difficult to explore different proteins associated with complex traits, such as RSA [168], proteins of different organelles, such as chloroplast [72], transmembrane [180], and mitochondria [35] and stress responses. 

Protein identification is key to improving abiotic stress tolerance and RSA in wheat, however, complete protein identification and analysis remains challenging. Different complex compounds in wheat tissues, such as polysaccharides, lipids, polyphenols, and other secondary metabolites disturb complete protein identification [181]. Protein extraction also depends on abundance, and relatively arbitrary parameters, such as molecular weight, charges, and chemical affinity of proteins. As a result, there is no universal extraction protocol for all proteins from different tissues [24]. In addition, the detection of low abundance proteins (which may have an important role in RSA and stress tolerance identification) during complete protein identification is challenging [35]. A label-free quantitative approach was expected to address this challenge, but low abundance protein identification remains inaccurate and protein coverage is also poor [182]. Furthermore, exploring the PTM of proteins and identifying common signaling pathways of multiple stress responses are challenging [24]. Conclusively, protein extraction and quantification methods need refining, and a complete protein database is required to explore wheat proteomics for RSA improvement and abiotic stress tolerance.

Despite having few limitations, proteomics is a promising field that can supplement the existing genomic knowledge in wheat to explore the mechanisms of genotype-environment interactions [183,184]. Furthermore, proteomics can help to understand PTMs [27]. Proteomic approaches allow the reconstruction of whole proteins under different stresses and identification pathways associated with individual stress [24]. Additionally, advanced label-free techniques can identify a range of proteins including low abundance proteins, at a lower cost, and more quick analysis than labeled techniques [185,186]. It also offers subcellular (e.g., chloroplast, grain nucleus) protein identification [72,187]. Furthermore, the genes encoding key DEPs can be used in marker-assisted breeding or genome-editing approaches for wheat RSA and stress tolerance improvement [72]. 

Genetic engineering has been used to develop stress tolerance in wheat since 1992 [188]. Currently, CRISPR-Cas9, a potential genome-editing technique has been used in wheat improvement [189,190,191,192,193], and it can be combined with proteomics to achieve higher genetic gains in wheat breeding programs. Currently, quantitative proteomics is used to study the effect of protein knockout using CRISPR-Cas [194]. During genome editing, CRISPR-Cas technology uses many proteomic methods to study protein–protein and protein–chromatin interactions [195]. As an example, affinity purification with MS (AP-MS) protein–protein interactions can be studied precisely and on a large scale [196]. Combining CRISPR-Cas and AP-MS solves the challenges of mislocalization of proteins and their non-endogenous binding during genome editing [195]. In wheat, subcellular protein identification has been explored by combining CRISPR-Cas as it allows the direct insertion of fluorescent tags into the protein-coding gene frame [191]. 

Moreover, proteomics is a powerful tool for detecting novel DEPs or DAPs in tolerant and sensitive genotypes, exploring the pathways of those proteins and understanding PTMs that play an important role in abiotic stress tolerance and RSA improvement in wheat. However, integrating proteomics with transcriptomics and metabolomics will help to explore the complete molecular basis of stress tolerance and RSA development in wheat.

## 7. Conclusions

Proteomics is an excellent molecular technique for explaining the molecular mechanisms of abiotic stress tolerance and plant parts, such as RSA development through the rigorous identification of proteins and their associated pathways. Over the decades, proteomics has advanced from conventional gel electrophoresis to label-free protein identification that covers more protein numbers. Proteomics has been used extensively to explore the molecular mechanisms of heat, salinity and drought stress tolerance in wheat. Under these stresses, photosynthesis, ROS scavenging and defense-associated proteins play a role in improving stress tolerance. Carbon metabolism proteins are involved in salinity and drought stress tolerance. Proteins, such as HSPs, peroxidases, GST and Trxs are also important for stress tolerance improvement. Seed storage proteins, such as gliadins play an important role in grain development and grain quality during stresses. Cell wall generation proteins, carbohydrate metabolism proteins, peroxidases, and a few defense proteins are associated with RSA growth and development. These proteins can be used as markers in marker-assisted breeding to select desired cultivars (tolerant) and genetic engineering can use candidate proteins to develop stress-tolerant wheat cultivars with improved RSA. However, challenges associated with the large and complex wheat genome, the lack of a wheat protein database, and identifying subcellar proteins and low abundance proteins using current proteomic tools, need to be addressed for a complete exploration of the molecular basis of stress tolerance and RSA development in wheat. 

## Data Availability

Not applicable.
